# Disparities in indication for primary cesarean delivery before and after implementation of quality initiatives

**DOI:** 10.1002/pmf2.70314

**Published:** 2026-05-23

**Authors:** Anna Marie Pacheco Young, Bethany Stetson, Nicola Lancki, Melissa Bak, Kaeli Heidenreich, Lane Patterson, Nigel Madden

**Affiliations:** ^1^ Department of Maternal Fetal Medicine Brown University/Women & Infants Providence Rhode Island USA; ^2^ Department of Maternal Fetal Medicine Ohio State University Columbus Ohio USA; ^3^ Department of Epidemiology Northwestern Feinberg School of Medicine Chicago Illinois USA; ^4^ Department of Internal Medicine Northwestern Feinberg School of Medicine Chicago Illinois USA; ^5^ Department of Obstetrics and Gynecology University of Pittsburgh Pittsburgh Pennsylvania USA; ^6^ Northwestern Feinberg School of Medicine Chicago Illinois USA; ^7^ Department of Maternal Fetal Medicine Beth Isreal Deaconess Medical Center Boston Massachusetts USA

**Keywords:** health disparities, indication for cesarean, NTSV cesarean delivery, primary cesarean delivery, quality improvement

## Abstract

**Background:**

Racial and ethnic disparities exist in the rates of and indication for cesarean delivery, prompting the implementation of quality initiatives designed to target the drivers of these inequities. Few studies have assessed the impact of these initiatives on reducing these disparities.

**Objective(s):**

To determine the rates of and indications for cesarean delivery before and after the implementation of perinatal quality improvement (QI) initiatives.

**Study design:**

Retrospective cohort study of 3629 nulliparous individuals with full‐term live‐born, singleton gestations in vertex position (NTSV) who delivered at a large academic tertiary care center in the United States from October 2019 through December 2022. Perinatal QI initiatives, designed to promote vaginal birth and improve equity in birth outcomes, were implemented during this period. This included the Promoting Vaginal Birth (PVB) initiative implemented in October 2020, followed by the Birth Equity (BE) initiative in May 2021. An interrupted time series analysis was performed to examine the relationship between the implementation of perinatal QI initiatives and trends in cesarean delivery rates. Distributions of demographic, clinical, and structural characteristics were examined by indication for cesarean and race and ethnicity. Multivariable multinomial logistic regression models assessed the independent association between race and ethnicity and indication for cesarean delivery after adjusting for maternal, perinatal, and system‐level risk factors before and after implementation of perinatal quality initiatives.

**Results:**

The rate of NTSV deliveries increased immediately following the start of quarter I (step‐change *β* = 3.6%, 95% confidence interval [CI], 1.4%–5.8%, *p* = 0.004). However, across the study period, the rate of NTSV cesarean deliveries decreased compared to the estimated trend prior to the initiatives for a difference in slope per quarter of −0.6% (95% CI, −1.0 to −0.1, *p* = 0.019). Non‐Hispanic (NH) Black pregnant people had the highest cesarean delivery rate: 29% compared to a range of 21%–23% in other racial and ethnic groups (*p* < 0.001). NH Black individuals had higher relative proportions of NTSV cesarean delivery for non‐reassuring fetal status as compared to NH White individuals (38.1% vs. 25.9%), and Hispanic individuals had higher relative proportions of NTSV cesarean delivery for arrest of dilation as compared to NH White individuals (33.4% vs. 24.5%). After adjusting for maternal, perinatal, and systems‐level risk factors, NH Black individuals had a higher risk of NTSV cesarean delivery for non‐reassuring fetal status compared to NH White pregnant people over the entire study period (relative risk ratio [RRR] 1.91, 95% CI, 1.27–2.86). The association between race and indication for cesarean delivery did not differ by the implementation period of QI initiatives.

**Conclusion(s):**

Quality initiatives to promote vaginal birth and birth equity may reduce the overall rate of NTSV cesarean delivery. However, additional efforts are necessary to understand and mitigate disparities in indication for cesarean delivery among minority pregnant individuals.

## 1 INTRODUCTION

Cesarean delivery has been associated with increased maternal morbidity and mortality [1]. While some cesarean deliveries are unavoidable, the primary cesarean delivery in nulliparas at term with singleton, vertex presenting (NTSV) pregnancies represents a subset of cesarean deliveries that is potentially more amenable to preventative measures. The World Health Organization (WHO) recommends that the rate of NTSV cesarean deliveries remains below 10%–15% [2]. Despite these goals, rates of cesarean delivery, including NTSV cesarean deliveries, have continued to rise in the United States [3].

It is well established that minority pregnant individuals experience higher rates of maternal mortality and morbidity, including higher rates of cesarean deliveries and their complications, as compared to their White counterparts [2, 3].  Studies have also shown that disparities exist in the rates of and indications for NTSV cesarean deliveries with non‐Hispanic (NH) Black and Hispanic pregnant individuals having higher odds of undergoing cesarean delivery for non‐reassuring fetal status (NRFS) [4, 5, 6].

Risk factors have been explored to explain the existing disparities including individual‐level factors such as maternal demographics, pre‐existing and pregnancy‐related medical comorbidities, and antenatal and perinatal complications, as well as wider systemic factors including general access to health care, existence and allocation of hospital resources, and discrimination and racism among health care institutions and providers [4, 5, 6].

The emphasis on system‐level factors has encouraged the implementation of many perinatal quality of care initiatives. A statewide network committed to improving maternal and neonatal health implemented the Promoting Vaginal Birth (PVB) initiative in October 2020 and the Birth Equity (BE) initiative in May 2021 with the goal of promoting equity among minority pregnant people during labor and delivery [7, 8, 9].

It is imperative to evaluate and monitor the effects of these initiatives to assess equity within our own institution and to assist in the development of model quality improvement (QI) initiatives for other hospitals. Therefore, our objective was to evaluate the association between maternal race and ethnicity and the rates of and indications for NTSV cesarean delivery before and after the implementation of perinatal QI initiatives.

## 2 MATERIALS AND METHODS

This was a retrospective cohort study of nulliparous pregnant people with live, term, singleton gestations in the vertex position who delivered at a large academic tertiary care center in the United States from October 2019 to December 2022. Gestational age at term was defined as greater than or equal to 37 weeks gestational age. All NTSV deliveries during this time were queried to evaluate the overall cesarean delivery rate, defined as the percentage of NTSV deliveries that occurred by cesarean delivery. The primary analysis, evaluating the indication for NTSV cesarean delivery, included only individuals who delivered by cesarean delivery. Patients under the age of 18 were excluded due to differing consent frameworks and distinct obstetric risk profiles.

**FIGURE 1:**
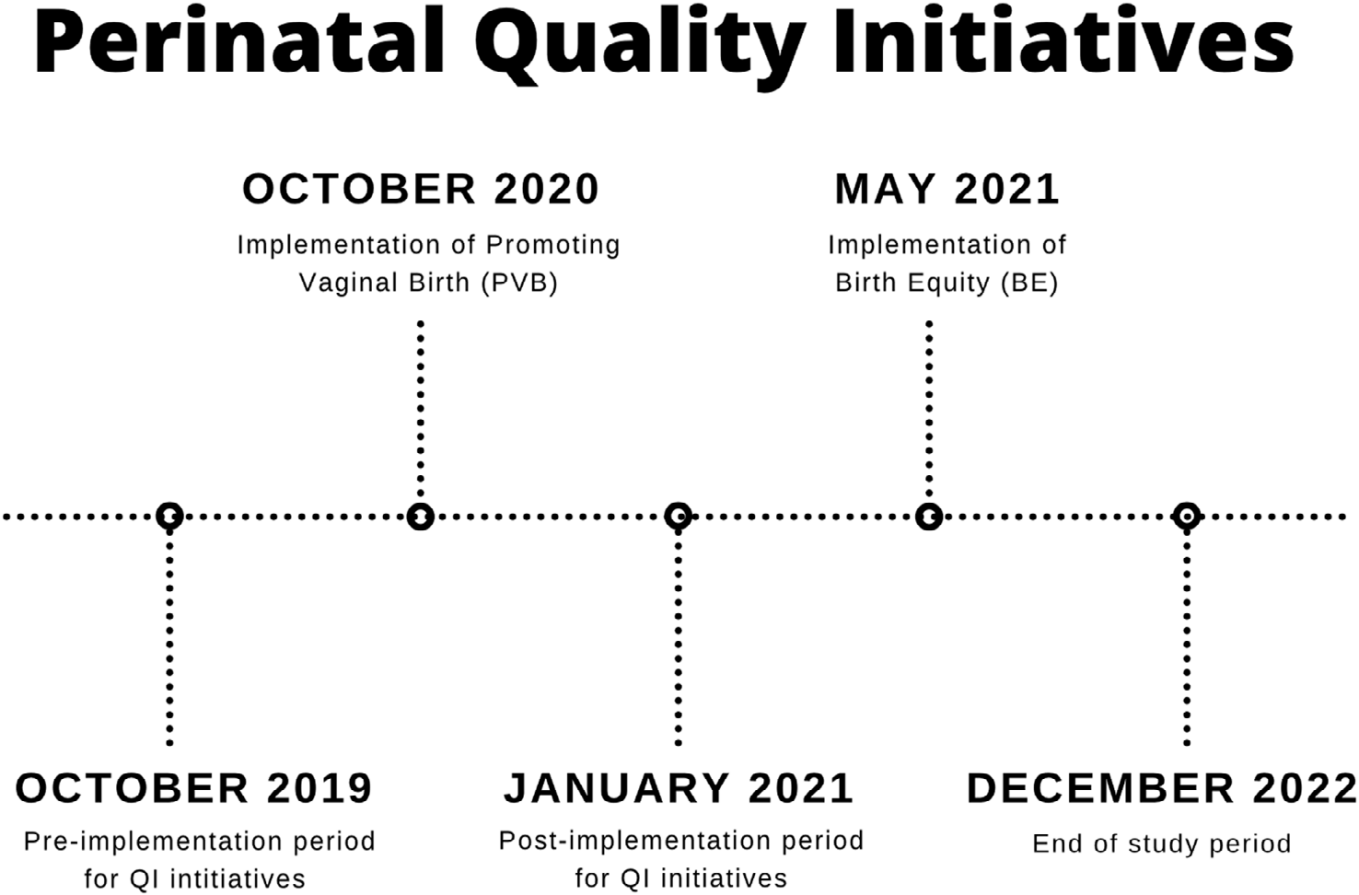
Timeline of study period and implementation of quality initiatives via our statewide quality collaborative. BE, Birth Equity; PVB, Promoting Vaginal Birth; QI, quality initiatives.

During the study period, the statewide collaborative and our institution implemented two separate QI initiatives, the PVB initiative and the BE initiative. These initiatives were meant to target all obstetric providers such as attendings, midwives, residents, and nursing staff (Figure 1) [8, 9].

The PVB initiative was implemented with the goal of reducing primary cesarean delivery rates through standardization of evidence‐based labor management practices. Key components included development and dissemination of standardized induction of labor protocols aligned with American College of Obstetricians and Gynecologists (ACOG) guidelines, reinforcement of active labor thresholds prior to diagnosis of arrest disorders, structured management algorithms for labor abnormalities, and educational sessions for clinicians involved in intrapartum care.

The PVB initiative began with working groups starting in October 2020 followed by implementation of pre‐cesarean delivery checklists and templates for documentation of indication for cesarean via the electronic medical record (EMR). Specifically, when documenting a need for NTSV cesarean delivery, providers submitted a checklist within a standardized dot phrase highlighting the specific criteria by which their patient met indication for cesarean delivery. These were later reviewed by members of the perinatal quality collaborative.

Staff guides for labor support were also provided throughout the unit. These interventions were intended to reduce potentially avoidable cesarean deliveries by promoting adherence to standardized labor management criteria. For example, reinforcement of active labor thresholds and guidance on labor progression were expected to reduce cesarean deliveries for arrest of dilation or arrest of descent by encouraging adequate time for labor progression. Similarly, standardized induction protocols were intended to reduce cesarean deliveries for failed induction by ensuring appropriate cervical ripening and duration of labor prior to cesarean delivery. Materials to support dissemination were provided to providers and nursing staff by the perinatal quality collaborative at our hospital.

Starting in June 2021, our department participated in periodic audit‐and‐feedback processes in which institutional NTSV cesarean delivery rates and indications were reviewed to identify opportunities for improvement. Staff benchmarking took place during this time period as a method of maintenance of set standards at the beginning of the initiative. This was primarily done by providing separate practice groups with their overall and individual physician cesarean delivery rates. Assessment of department uptake and coverage of interventions was performed by a member of the perinatal quality collaborative using the PVB Monthly Hospital Level Data Form [10]. This form assessed structured measures across the hospital for providers and nursing staff, including but not limited to cesarean decision checklists using ACOG/ SMFM labor guidelines, standardized patient education, sharing of provider‐level data and percentage of providers and nursing staff receiving standardized education regarding labor management, and decision huddles. More importantly, it ensured ongoing engagement of clinical staff in QI efforts.

The BE initiative, beginning in May 2021, had elements that overlapped with PVB. Starting again with working groups and department‐wide education, it included several institutional changes to address disparities in maternal health outcomes. This included implementation of EMR‐based universal social determinants of health (SDOH) screening with linkage to appropriate resources, ensuring accurate documentation of self‐reported race and ethnicity, review of maternal health quality data to identify disparities and address opportunities for improvement, engagement of patients to provide input on QI efforts through patient surveys, standardization of patient discharge education, and implementation of implicit bias and respectful care education for providers and staff.

During our study period, in May 2021, SDOH screening was integrated into the EMR with linkage to a personalized community platform that connects patients to community resources based on their responses. To facilitate better and more respectful care, patient advisors were recruited, and respectful care practice posters were displayed around the labor and delivery unit. Patient advisors provided personal experiences as a mode of feedback for the quality of care being provided and inspired new ideas for quality‐based interventions [11]. Respectful care practices included but were not limited to recognizing how past healthcare experiences could affect how patients felt during their labor and delivery, listening to ensure the patient felt heard, partnering together for medical decision‐making, and valuing personal boundaries [12]. These interventions were designed to address structural contributors to disparities in maternal outcomes rather than labor management practices.

Given our primary objective to evaluate for changes in rates and indication for NTSV cesarean deliveries, by race and ethnicity, we chose the PVB initiative as the core initiative for the analysis. Therefore, the period before quality changes was defined as October 2019 through October 2020 and post quality changes as January 2021 through December 2022. A 2‐month time gap after initiation of the PVB initiative was used to allow for the ramp up of these changes after initial implementation in our regression analyses (Figure 1).

A query of the EMR record identified eligible patients. Variables that could not be abstracted reliably through the data query, or that were missing or inconsistent, were manually reviewed and abstracted by the research team. Charts were randomly selected for review to ensure consistency by a senior member of the research team.

We first performed a broad analysis of the rates of NTSV deliveries that were completed by cesarean delivery and the indications for NTSV cesarean deliveries over our study period in order to understand the general trends in relation to these quality initiatives. Primary exposure was self‐reported race and ethnicity. Considering race to be a social rather than biologic construct, included patients were categorized by self‐identified race and ethnicity including Asian, Hispanic, NH Black, NH White, and other or unknown. Data regarding self‐identified race and ethnicity were gathered by nursing staff using institution‐wide standardized screening on admission and by patient verbal report.

Our primary outcome was indication for NTSV cesarean delivery. Cesarean delivery indications, as defined by ACOG, included arrest of descent, arrest of dilation, failed induction, NRFS, and abnormal placentation (i.e., placenta previa) or other (e.g., prior uterine surgery, elective, and suspected macrosomia) [13]. Other indications for primary cesarean such as abnormal placentation, prior uterine surgery, elective or maternal request, or suspected macrosomia were chosen as a bundled category to serve as our referent. This decision was made with the understanding that these NTSV cesarean deliveries were not exposed to our hypothesized risk factors or possible confounders since they were scheduled based on a medical indication.

Additional maternal characteristics abstracted included demographic details, pre‐pregnancy body mass index (BMI), and maternal medical comorbidities including chronic hypertension, pregestational diabetes, gestational diabetes, and pregnancy‐induced hypertension. Perinatal and delivery characteristics included diagnosis of oligohydramnios, placental abruption, type of labor (induction of labor vs. spontaneous labor), and gestational age at delivery. System‐level factors included insurance status (public vs. private), day of the week in which the delivery occurred (week night vs. weekend), time of delivery (day vs. night shift), and attending physician practice type (private vs. academic).

### 2.1 Statistical analyses

To examine trends in overall rates of NTSV deliveries and indications for cesarean deliveries, quarterly proportions of NTSV deliveries by cesarean delivery and the relative proportion of each indication for NTSV cesarean delivery over time were inspected graphically. We used an interrupted time series design with the start of the PVB initiative as the intervention, examining the immediate effect and effect over time on proportion of NTSV deliveries by cesarean delivery using a generalized linear regression model with an autoregressive residual correlation structure. Given that there were only four data points in the pre‐intervention time period with the use of quarterly proportions (October 2019–October 2020), the pre‐intervention slope estimation was extended to January 2019 to improve stability of trend estimation for the time series analysis. Residual autocorrelation was assessed by examining the plot of residuals and plot of the partial‐autocorrelation function and Durbin–Watson test statistic. The analytic cohort for the primary regression analyses spanned October 2019 through December 2022. This more stringent time window was used mainly given the amount of manual chart abstraction that was necessary for some of our more specific variables.

For the primary analysis examining indications for NTSV cesarean delivery, differences in distributions of demographic, clinical, and system‐level characteristics by indication for NTSV cesarean delivery and race and ethnicity were examined using chi‐square, Fisher's exact, or analysis of variance tests. Plots of the observed relative quarterly proportions of each indication for NTSV cesarean delivery over time by each racial and ethnic group were used to examine trends.

To evaluate the independent association between race and ethnicity and differences in indications for NTSV cesarean delivery, we used multivariable multinomial logistic regression models estimating odds of indication for NTSV delivery adjusted for maternal, perinatal, and systems‐level risk factors with the 2‐month exclusionary period to account for a transitionary period after implementation of PVB. Confounding variables included obesity (defined as pre‐pregnancy BMI > 30), diagnosis of any hypertensive disorder, any diabetes, oligohydramnios, placental abruption, gestational age, insurance status, day of the week in which the delivery occurred, shift during which the delivery occurred, and attending practice type. We did not consider assisted reproducive technology or regional anesthesia in our analysis. Gestational weight gain was not calculated. Covariates were selected a priori based on literature and conceptual causal framework with which we believed we achieved saturation [4, 5]. Other indications for cesarean, such as abnormal placentation, was the referent indication in all models. Finally, we examined if the QI initiative modified an association between indication for cesarean delivery and race and ethnicity by adding an interaction term between race and ethnicity and the pre‐ versus post‐PVB period. All analyses were performed in R version 4.1.3. Statistical significance was considered a *p*‐value of < 0.05.

## 3 RESULTS

**FIGURE 2:**
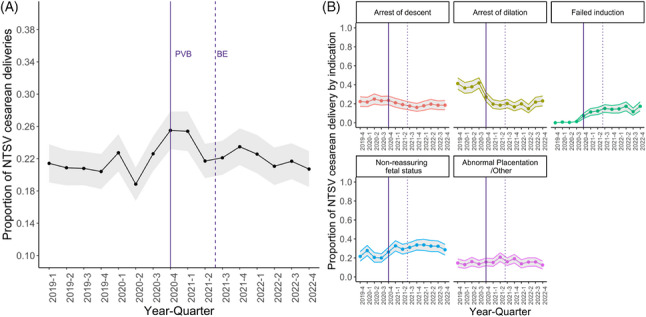
Proportion of overall Nulliparous Term Singleton Vertex (NTSV) deliveries by cesarean delivery (A) and indications for NTSV cesarean deliveries over time (B). Solid line indicates implementation of Promoting Vaginal Birth (PVB). Dotted line indicates implementation of Birth Equity (BE). Indications for cesarean delivery are as defined by the American College of Obstetricians and Gynecologists (ACOG). Includes data over the entire study period.

The overall NTSV cesarean delivery rate during the entire study period was 21.3%. Figure 2A shows the proportion of NTSV deliveries by cesarean with indicators at the start of PVB. Overall proportion of NTSV cesarean deliveries decreased after PVB with a slight uptrend following BE. Despite the uptick, the proportion of NTSV cesarean deliveries fell to less than the proportion prior to implementation of PVB. Figure 2B shows the relative proportion of all NTSV cesarean deliveries by indication over time. In general, rates of NTSV cesarean delivery for arrest of dilation decreased, whereas rates of NTSV cesarean delivery for failed induction and NRFS increased following PVB implementation starting in October 2020. The apparent absence of documented failed induction prior to Q4 2020 reflects a documentation change concurrent with PVB implementation. Prior to standardization of cesarean indication entry in the EMR, failed induction cases were frequently categorized under arrest disorders. Thus, the observed increase likely reflects improved classification rather than a true clinical increase.

**FIGURE 3:**
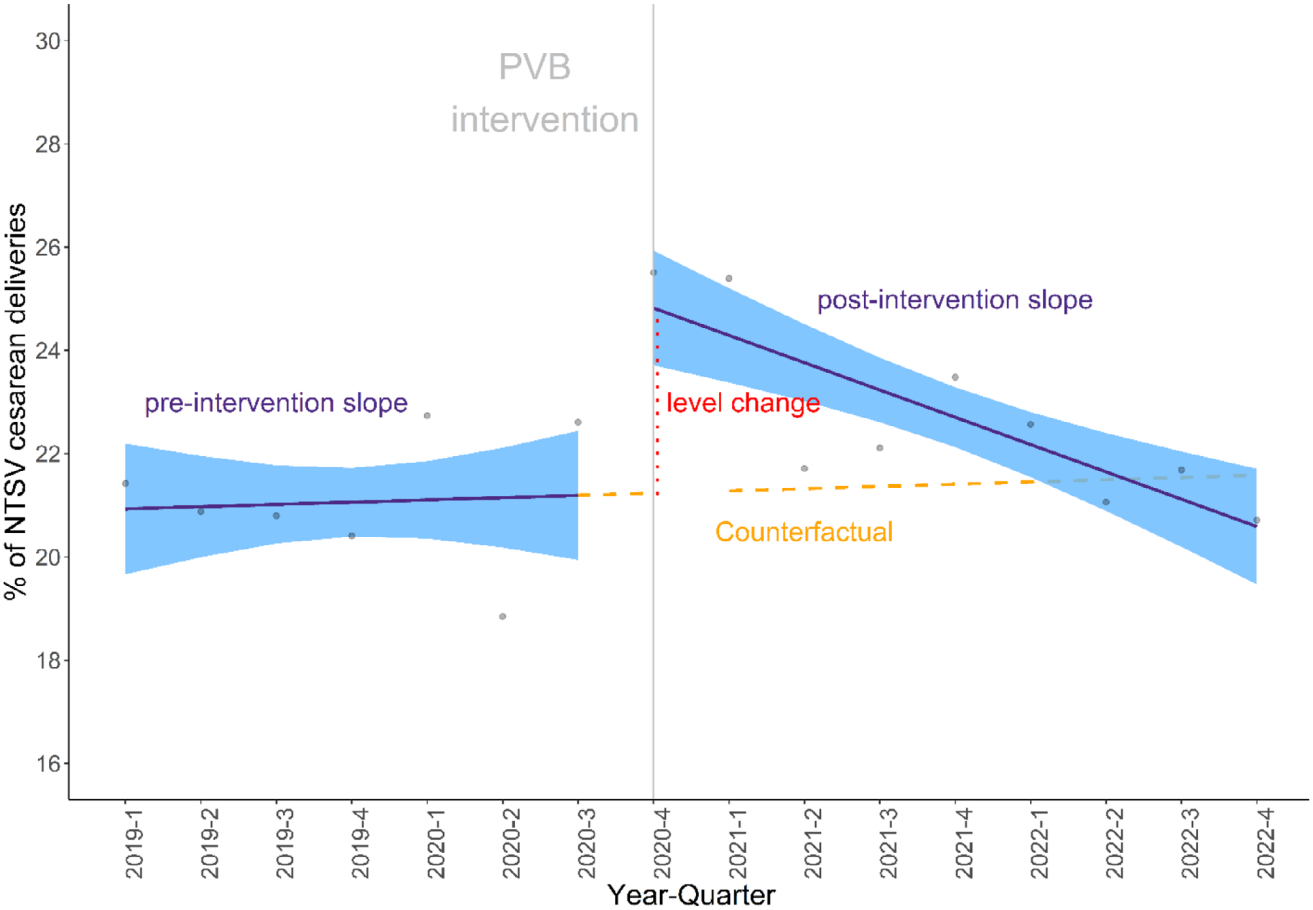
Time series analysis of overall proportion of Nulliparous Term Singleton Vertex (NTSV) cesarean deliveries before and after implementation of Promoting Vaginal Birth (PVB) based on quarterly data. The gray dots are the observed monthly proportions of cesarean pre‐ and postquality initiatives. The gray line represents implementation of PVB. The solid purple lines and blue shaded region show the model estimated rates and 95% confidence intervals of cesarean deliveries pre‐and post‐intervention. Prior to the intervention, the rate of cesareans appeared steady. The red dotted line indicates the level change in the rate of cesarean deliveries at the start of the intervention. The orange dotted line highlights the counterfactual, or the expected trend without implementation of PVB.

Figure 3 shows the time series analysis examining the effect of PVB on the proportion of NTSV deliveries by cesarean delivery over the entire study period. The orange line represents the counterfactual, which is the expected trend based on the pre‐intervention data. The intercept shows the estimated rate at the start of the study period was 20.9% (95% CI, 19.1–22.6). The quarterly trend prior to PVB was close to zero, indicating there was not a significant change in rates prior to the initiative (*β* = 0.04%, 95% CI, −0.4 to 0.4, *p*‐value = 0.814). The proportion of NTSV deliveries by cesarean delivery increased immediately after PVB (*β* = 3.6%, 95% CI, 1.4–5.8, *p* = 0.004) but decreased quarterly thereafter by a rate of change of minus 0.6% per quarter. This negative rate of change was significantly lower than the estimated slope prior to the initiative (*p*‐value = 0.019). Given lack of adequate sample size and large confidence intervals, we were not able to perform stratified time series analysis by race and ethnicity, however raw proportion of NTSV cesarean delivery rates stratified by race and ethnicity is shown in Figure S1.

The study population for the primary analysis included 3629 pregnant individuals who had an NTSV cesarean delivery. Of these individuals, 1852 (51.0%) identified as NH White, 431 (11.9%) as NH Black, 545 (15.0%) as Hispanic, and 341 (9.4%) as Asian. There were differences in the distributions of maternal, perinatal, and structural characteristics by race and ethnicity (Table 1). NH Black and Hispanic individuals were younger at delivery and a greater proportion were single and obese as compared to NH White individuals. NH Black individuals also had the highest proportions of chronic and pregnancy‐related medical comorbidities, including chronic hypertension, pregestational diabetes, pregnancy‐induced hypertension, and oligohydramnios. Asian individuals had greater proportions of gestational diabetes and pregestational diabetes. With regard to systems‐level factors, a greater proportion of NH Black and Hispanic individuals had public insurance and were delivered by an academic practice. Finally, NH Black, Hispanic, and Asian individuals had a greater proportion of residents involved in their care.

**TABLE 1:** Maternal, perinatal, and systems‐level characteristics stratified by race and ethnicity.

Characteristic	Overall (*N* = 3629)	NH white, *n* = 1852 (51.0)	NH black, *n* = 431 (11.9)	Hispanic, *n* = 545 (15.0)	Asian, *n* = 341 (9.4)	Other/ unknown, *n* = 460 (12.7)	*p* value
Advanced maternal age	1431 (39.4)	810 (43.7)	134 (31.1)	151 (27.7)	151 (44.3)	185 (40.2)	<0.001
Partnered	2936 (80.9)	1697 (91.6)	194 (45.0)	351 (64.4)	312 (91.5)	382 (83.0)	<0.001
Smoke exposure	407 (11.2)	237 (12.8)	48 (11.1)	51 (9.4)	28 (8.2)	43 (9.3)	0.024
Obese (BMI > 30 kg/m^2^)	2206 (61.6)	1035 (56.8)	343 (80.0)	411 (76.1)	160 (47.5)	257 (56.2)	<0.001
Gestational age (weeks)	39.5 (1.1)	39.5 (1.1)	39.3 (1.2)	39.6 (1.1)	39.5 (1.0)	39.5 (1.1)	0.006
Female neonatal sex	1547 (42.7)	765 (41.4)	202 (46.9)	239 (43.9)	145 (42.5)	196 (42.6)	0.316
Birthweight (g)	3441 (491)	3480 (481)	3296 (475)	3461 (502)	3376 (508)	3442 (498)	<0.001
Polyhydramnios	139 (3.8)	75 (4.0)	15 (3.5)	18 (3.3)	7 (2.1)	24 (5.2)	0.190
Oligohydramnios	55 (1.5)	21 (1.1)	8 (1.9)	17 (3.1)	5 (1.5)	4 (0.9)	0.012
Placental abruption	53 (1.5)	30 (1.6)	3 (0.7)	8 (1.5)	8 (2.3)	4 (0.9)	0.296
Chorioamnionitis	727 (20.0)	308 (16.6)	89 (20.6)	138 (25.3)	99 (29.0)	93 (20.2)	<0.001
Type of labor							0.019
Induction	2139 (58.9)	1079 (58.3)	257 (59.6)	334 (61.3)	191 (56.0)	278 (60.4)	
Spontaneous	1044 (28.8)	521 (28.1)	113 (26.2)	158 (29.0)	120 (35.2)	132 (28.7)	
Chronic hypertension	138 (3.8)	58 (3.1)	40 (9.3)	17 (3.1)	8 (2.3)	15 (3.3)	<0.001
Pregnancy‐induced hypertension	859 (23.7)	416 (22.5)	138 (32.0)	142 (26.1)	63 (18.5)	100 (21.7)	<0.001
Preeclampsia							<0.001
Preeclampsia without severe features	158 (4.4)	61 (3.3)	28 (6.5)	36 (6.6)	14 (4.1)	19 (4.1)	
Preeclampsia with severe features	365 (10.1)	164 (8.9)	69 (16.0)	64 (11.7)	30 (8.8)	38 (8.3)	
Pre‐gestational diabetes	61 (1.7)	28 (1.5)	10 (2.3)	10 (1.8)	1 (0.3)	12 (2.6)	0.098
Gestational diabetes	268 (7.4)	105 (5.7)	39 (9.0)	36 (6.6)	50 (14.7)	38 (8.3)	<0.001
Publicly insured	403 (11.1)	31 (1.7)	136 (31.6)	187 (34.3)	19 (5.6)	30 (6.5)	<0.001
Weekday delivery (vs. weekend)	2229 (61.4)	1149 (62.0)	264 (61.3)	316 (58.0)	204 (59.8)	296 (64.3)	0.282
Day shift delivery (vs. night shift)	1132 (31.2)	577 (31.2)	139 (32.3)	161 (29.5)	113 (33.1)	142 (30.9)	0.821
Attending practice model							<0.001
Private	2948 (81.2)	1614 (87.1)	275 (63.8)	405 (74.3)	282 (82.7)	372 (80.9)	
Academic	453 (12.5)	144 (7.8)	121 (28.1)	80 (14.7)	42 (12.3)	66 (14.3)	
Unknown	228 (6.3)	94 (5.1)	35 (8.1)	60 (11.0)	17 (5.0)	22 (4.8)	
Training of provider							<0.001
Resident	2595 (71.6)	1283 (69.4)	323 (74.9)	399 (73.5)	264 (77.6)	326 (70.9)	
Fellow	69 (1.9)	28 (1.5)	18 (4.2)	13 (2.4)	1 (0.3)	9 (2.0)	
Attending	959 (26.5)	538 (29.1)	90 (20.9)	131 (24.1)	75 (22.1)	125 (27.2)	
Indication for CS							<0.001
Arrest of descent	739 (20.4)	435 (23.5)	38 (8.8)	94 (17.2)	70 (20.5)	102 (22.2)	
Arrest of dilation	948 (26.1)	454 (24.5)	111 (25.8)	182 (33.4)	83 (24.3)	118 (25.7)	
Failed induction	343 (9.5)	167 (9.0)	49 (11.4)	61 (11.2)	31 (9.1)	35 (7.6)	
Non‐reassuring fetal status	1032 (28.4)	479 (25.9)	164 (38.1)	143 (26.2)	111 (32.6)	135 (29.3)	
Abnormal placentation/other	567 (15.6)	317 (17.1)	69 (16.0)	65 (11.9)	46 (13.5)	70 (15.2)	

*Note*: Spontaneous labor is defined as any patient who presented with contractions and cervical change and or rupture of membranes and meeting indication for augmentation. Induction of labor was defined as any scheduled elective induction or medically indicated induction. Data are presented as *N* (%) or mean (standard deviation). Frequency (%) for categorical/dichotomous variables, and mean (SD) for continuous variables. Missing data (obese: *n* = 45, neonatal sex: *n* = 2, birthweight: *n* = 17, insurance: *n* = 1, time of delivery: *n* = 3, training of provider: *n* = 6). It includes data over the entire study period.

Abbreviations: BMI, body mass index; NH, non‐Hispanic.

Overall, 28.4% of individuals had an NTSV cesarean delivery for NRFS, 26.1% for arrest of dilation, 20.4% for arrest of descent, 9.5% for failed induction of labor, and 15.6% for abnormal placentation or other indication (Table S1). When examining distributions of indications over the entire study period, NH Black individuals had a higher proportion of NTSV cesarean delivery for NRFS and failed induction but a lower proportion of arrest of descent compared to other groups. Hispanic individuals had a higher proportion of NTSV cesarean delivery for arrest of dilation and failed induction and Asian individuals had a higher proportion of NTSV cesarean delivery for NRFS (Table S1). Additional maternal, perinatal, and system‐level characteristics by indication for NTSV cesarean delivery are shown in Table S2.

**FIGURE 4:**
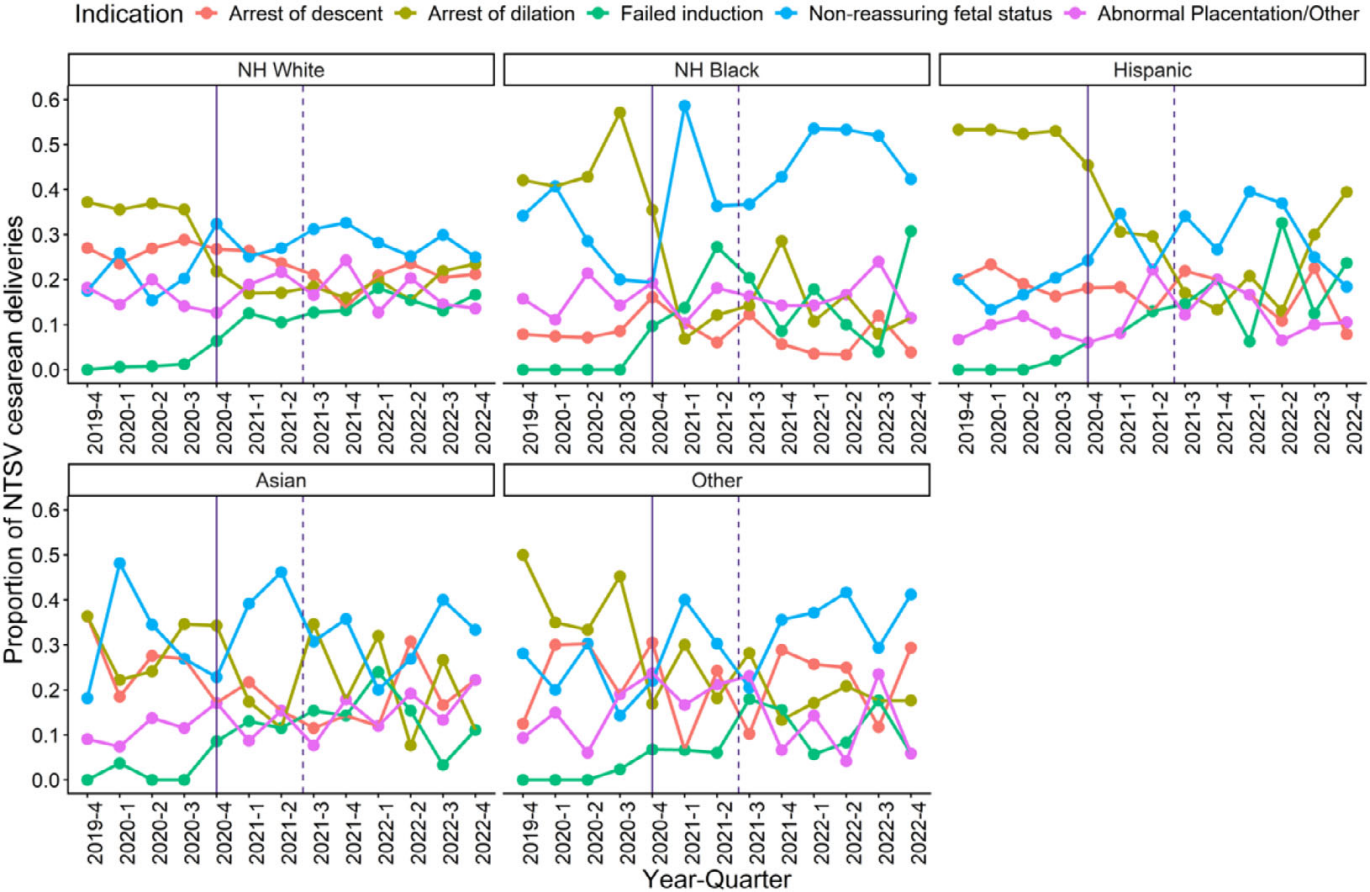
Quarterly proportion of indication for Nulliparous Term Singleton Vertex (NTSV) cesarean delivery by race and ethnicity. Solid line indicates implementation of Promoting Vaginal Birth (PVB). Dotted line indicates implementation of Birth Equity (BE). Indications for cesarean delivery are as defined by the American College of Obstetricians and Gynecologists (ACOG). Includes data over the entire study period.

The proportion of NTSV delivery by indication for each race and ethnicity is shown in Figure 4 and or Figure S2. Across the study period, deliveries for failed induction increased across all racial and ethnic groups. Additionally, deliveries for arrest of descent and abnormal placentation or other indications remained relatively stable across racial and ethnic groups. In NH Black pregnant people, deliveries for arrest of dilation, the most common indication prior to PVB, decreased after implementation. In this population, the proportion of deliveries for NRFS, which had been declining prior to PVB, increased immediately after implementation and was the most common indication for NTSV cesarean delivery in this group at the end of the study period. In Hispanic pregnant people, NTSV cesarean delivery for arrest of dilation similarly decreased substantially following implementation. Similar trends of decreasing deliveries for arrest of dilation and increasing deliveries for NRFS were seen in NH White pregnant people, however these changes were more subtle.

NH Black pregnant people had a lower risk of NTSV cesarean delivery for arrest of descent (relative risk ratio [RRR] 0.37, 95% CI, 0.22–0.63) and a higher risk of NTSV cesarean delivery for NRFS (RRR 1.91, 95% CI, 1.27–2.86) compared to NH White pregnant people after adjusting for confounders (Table 2). Hispanic pregnant people had a greater risk of NTSV cesarean delivery for arrest of dilation (RRR 1.40, 95% CI, 0.96–2.05), whereas Asian pregnant people had a greater risk of NTSV cesarean delivery for NRFS (RRR 2.01, 95% CI, 1.31–3.08) as compared to NH White pregnant people when adjusting for confounders (Table 2). We did not observe an association between Hispanic ethnicity and risk of NTSV cesarean for arrest of dilation in our unadjusted analysis (Table S3).

**TABLE 2:** Adjusted multinomial regression model estimates for the association of race and ethnicity and indication for NTSV cesarean delivery.^a^, ^b^

	Indication for cesarean			
	Arrest of descent RRR (95% CI)	Arrest of dilation RRR (95% CI)	Failed induction RRR (95% CI)	Non‐reassuring fetal status RRR (95% CI)
NH White	Ref	Ref	Ref	Ref
NH Black	**0.37 (0.22, 0.63)**	1.08 (0.71, 1.66)	1.07 (0.64, 1.79)	**1.91 (1.27, 2.86)**
Hispanic	0.80 (0.53, 1.21)	**1.40 (0.96, 2.05)**	1.31 (0.82, 2.08)	1.04 (0.70, 1.53)
Asian	1.37 (0.87, 2.15)	1.56 (0.99, 2.43)	1.62 (0.93, 2.81)	**2.01 (1.31, 3.08)**
Other or unknown	1.16 (0.78, 1.71)	1.35 (0.92, 1.99)	1.02 (0.61, 1.68)	**1.58 (1.08, 2.31)**

*Note*: Bold values indicate significant results. Excludes 2‐month transition period.

Abbreviations: CI, confidence interval; NH, non‐Hispanic; NTSV, Nulliparous Term Singleton Vertex; RRR, relative risk ratio.

^a^ Indications for cesarean delivery are as defined by the American College of Obstetricians and Gynecologists.

^b^ Adjusted for age at delivery, BMI at delivery, hypertension, pregestational and gestational diabetes, oligohydramnios, placental abruption, gestational age, insurance, day of week, time of delivery, and practice model of attending.

In the adjusted model evaluating the association between race and ethnicity with indication for NTSV cesarean delivery before and after PVB, there was insufficient evidence to suggest that the initiatives altered this association (*p* for interaction = 0.322). Additional maternal, perinatal, and structural characteristics of participants pre‐ and postimplementation period with and without the transition months are included in Tables S4 and S5.

## 4 DISCUSSION

After implementation of the PVB quality initiative, we saw an initial spike in the overall rate of NTSV deliveries by cesarean followed by a significant decrease in the rate of NTSV cesarean deliveries compared to expected if the initiative had not been implemented. The distribution of indications for NTSV cesarean deliveries changed with this initiative as well, with a decrease in deliveries for arrest of dilation and increase in deliveries for failed induction and NRFS. There were significant differences between racial and ethnic groups in the indication for NTSV cesarean delivery, however implementation of the PVB initiative did not alter these disparities.

Our results are consistent with prior published literature. In a secondary analysis of a large, multicenter trial, among over 5000 nulliparous pregnant people, NH Black and Hispanic nulliparous people were more likely to undergo cesarean delivery as compared to their NH White counterparts, with higher rates of cesarean delivery for NRFS [4]. Another study similarly showed that NH Black pregnant people had higher odds of cesarean delivery for NRFS and that Asian and Hispanic pregnant people had higher odds of cesarean delivery for failure to progress [5].

Thus far, few studies have assessed the impact of implementation of quality initiatives on cesarean delivery. An observational study observed rates of NTSV cesarean delivery decreased over time after implementation of quality initiatives such as intensive data review, education, and mentoring [14]. Another study assessing hospital wide implementation of quality initiatives, including didactic and simulation education for providers, chart auditing, and changes in documentation of fetal heart tracing abnormalities, showed that their raw rate of cesarean delivery decreased from 35% to 21% [15]. These studies looked at the general patient population and did not assess racial disparities or changes in indication for cesarean delivery.

Our data similarly suggest that implementation of initiatives, such as PVB, may reduce overall NTSV cesarean deliveries and more appropriately categorize the indications for NTSV cesarean delivery. However, our study did not show a significant reduction in racial and ethnic disparities in indication for cesarean delivery. It is possible that we did not allow enough time for these interventions to have a full effect and that certain interventions that may have facilitated eliminating disparities such as patient surveys and required implicit bias training were not implemented until much later. However, it is also possible the interventions implemented are not targeting the appropriate drivers of these disparities. In a prior study at our institution that demonstrated higher rates of cesarean delivery for NRFS in NH Black pregnant people, the authors observed that numerous maternal, perinatal, and systemic factors could not explain the racial/ethnic disparities in cesarean delivery rates or indications for cesarean delivery [6].

The reasons for these disparities remain understudied, challenging to measure, and likely include factors that act on the patient, clinician and system level. One such system‐level factor may be the impact of structural racism and discrimination. Structural racism in both the past and present has resulted in deeply rooted mistrust in healthcare institutions and providers, particularly with racially discordant care, as well as a general increase in discrimination‐related stress during health care provision [16, 17]. Though structural racism is fortunately rarely overt in modern medical practice, it may also act subconsciously through implicit bias. Despite implementation of respectful care practices as part of the BE initiative overlapping with PVB during our study period, implicit bias is complex and may extend beyond the scope of this intervention.

Implicit bias may influence outcomes through various mechanisms including educational bias among trainees, provider–patient communication style, assessment of the cause and severity of symptoms, and clinical decision‐making and treatment recommendations [16, 17]. Specifically, the decision to proceed with cesarean delivery for NRFS often occurs in time‐sensitive clinical situations and requires interpretation of fetal heart rate tracings that may be inherently subjective. Prior work has suggested that variation in interpretation of category II tracings and thresholds for intervention may contribute to differences in cesarean delivery patterns [18, 19]. The acute atmosphere and subjective nature of the labor and delivery unit, particularly with regard to the decision to proceed with cesarean delivery, makes these interactions especially prone to implicit bias and efforts to directly address this should be emphasized [4, 6, 20, 21].

More recently, our state initiated mandatory implicit bias training for all hospitals, with a requirement for physicians to complete this training [8, 9]. Interventions such as these have the potential to address underlying social and racial mechanisms that drive disparities in cesarean delivery and need to be further studied. The BE initiative included components such as implicit bias training, standardized documentation of race and ethnicity, and review of quality metrics stratified by race and ethnicity. Our study period overlapped with the early implementation of some components of the BE initiative, but not all elements were in place during the study window. Notably, the statewide requirement for provider completion of implicit bias training was implemented later, beginning in January 2023, after the study period had concluded. This could also explain why we did not observe a reduction in disparity after implementation of PVB and the beginning stages of BE [22].

Implementation science frameworks that evaluate the uptake and impact of quality initiatives across different patient populations may help identify whether interventions are equitably improving care. Further research is needed to better understand the mechanisms driving disparities in cesarean delivery indications and to design targeted interventions that address both overall obstetric outcomes and equity in maternal care. This includes but is not limited to implicit bias, patient–provider communication, and structural factors that may influence decision‐making in the labor and delivery setting. One such strategy may incorporate prioritization of racial, ethnic, and language concordant care, which can help mitigate disparities. Health systems should continue to strengthen their diversity and inclusion initiatives, and actively recruit providers that reflect their patient population [23, 24, 25].

There are several strengths to our study. This study is one of the first to assess the impact of hospital‐wide quality initiatives on racial disparities in *indication* for NTSV cesarean delivery in collaboration with a statewide perinatal quality collaborative. This assessment provides an outline for how other hospitals can develop a monitoring plan for similar QI interventions. Our analysis of the overall rate of cesarean delivery before and after implementation of QI initiatives is novel because we used a time series analysis to compare observed rates with a counterfactual, or predicted trend, based on prior data. Additionally, our patient population includes a high‐risk cohort of patients with diverse backgrounds, which is important for disparities research and enhances generalizability.

Our results must be interpreted within the context of several limitations. We were not able to perform more granular analyses by individual cesarean delivery indication or by each racial and ethnic group due to limitations of sample size. There were several quality initiatives, most notably the BE initiative, that were implemented at various times within our study period and may have also contributed to the relationships observed. The collaborative learning structure of this quality initiative, including clinician education sessions, periodic audit‐and‐feedback of institutional performance metrics, and provider benchmarking, likely contributed to the gradual adoption of standardized labor management practices, which may have also impacted trends over time.

While this does make interpretation of the results more challenging, our primary outcome metric of indication for NTSV cesarean delivery is likely to be most influenced by the PVB initiative, around which we based our analysis. Additionally, as system‐level quality initiatives are frequently overlapping or implemented in phases in actual clinical practice, this challenge likely represents a common occurrence in quality research. It is possible that during this study period there may have been other unmeasured confounders at play, including events across time that may have influenced our findings. For example, our study period overlapped with the COVID‐19 pandemic, which according to a large systematic review was associated with an increased rate of cesarean delivery [26]. However, because the pandemic occurred across pre‐ and post‐intervention periods, its potential effects would likely influence both time periods. Finally, we chose a 2‐month time gap to allow for ramp up of the PVB changes prior to the post‐implementation study period; however, it is possible that this time gap was not sufficient.

While quality initiatives may help reduce NTSV cesarean delivery rates, our study highlights the challenges in implementing effective programs that address the racial and ethnic disparities in rates of and indications for primary cesarean delivery among nulliparous individuals. Future work is needed to better understand why these disparities exist and how to best implement QI initiatives to promote equity.

## CONFLICT OF INTEREST STATEMENT

The authors declare no conflicts of interest.

## ETHICS STATEMENT

This study was approved by the Institutional Review Board of Northwestern Feinberg School of Medicine (IRB# STU00218308).

## Supporting information



Supporting information

Supporting information
