# Supplementary material for: Disparities in indication for primary cesarean delivery before and after implementation of quality initiatives
Source: Pregnancy (Hoboken). 2026 May 23;2(3):e70314. doi: 10.1002/pmf2.70314 (PMC13344202; doi:10.1002/pmf2.70314)
Supplement: Supplementary file 1 [file PMF2-2-e70314-s001.docx]

**Supplemental Figure 1.** Quarterly proportion NTSV cesarean delivery rates by race and ethnicity. Solid line indicates implementation of PVB. Dotted line indicates implementation of BE.^1^

NTSV = Nulliparous Term Singleton Vertex; PVB = Promoting vaginal birth; BE = Birth Equity

^1^Indications for cesarean delivery are as defined by the American College of Obstetricians and Gynecologists

Includes data over the entire study period

**Supplemental Figure 2.** Quarterly proportion of indication for NTSV cesarean delivery by race and ethnicity. Dashed line with dots indicates implementation of PVB. Dashed line indicates implementation of BE.^1^

NTSV = Nulliparous Term Singleton Vertex; PVB = Promoting vaginal birth; BE = Birth Equity

^1^Indications for cesarean delivery are as defined by the American College of Obstetricians and Gynecologists (ACOG)

Includes data over the entire study period.
