# Supplementary material for: Disparities in indication for primary cesarean delivery before and after implementation of quality initiatives
Source: Pregnancy (Hoboken). 2026 May 23;2(3):e70314. doi: 10.1002/pmf2.70314 (PMC13344202; doi:10.1002/pmf2.70314)
Supplement: Supplementary file 2 [file PMF2-2-e70314-s002.docx]

| **Supplemental Table 1.** Indication for NTSV cesarean delivery by race and ethnicity^1^ | | | | | | |  |
| --- | --- | --- | --- | --- | --- | --- | --- |
|  | **Overall** N=3629 | **NH white**  N=1852  (51.0) | **NH black** N=431  (11.9) | **Hispanic** N=545  (15.0) | **Asian**  N=341  (9.4) | **Other or unknown** N=460  (12.7) | **p-value** |
|  |  |  |  |  |  |  | <0.001 |
| Non-reassuring fetal status | 1032 (28.4) | 479 (25.9) | 164 (38.1) | 143 (26.2) | 111 (32.6) | 135 (29.3) |  |
| Arrest of dilation | 948 (26.1) | 454 (24.5) | 111 (25.8) | 182 (33.4) | 83 (24.3) | 118 (25.7) |  |
| Arrest of descent | 739 (20.4) | 435 (23.5) | 38 (8.8) | 94 (17.2) | 70 (20.5) | 102 (22.2) |  |
| Failed induction | 343 (9.5) | 167 (9.0) | 49 (11.4) | 61 (11.2) | 31 (9.1) | 35 (7.6) |  |
| Abnormal placentation/other | 567 (15.6) | 317 (17.1) | 69 (16.0) | 65 (11.9) | 46 (13.5) | 70 (15.2) |  |

NTSV = Nulliparous Term Singleton Vertex, NH = non-Hispanic

Data presented as N (%)

^1^Indications for cesarean delivery are as defined by the American College of Obstetricians and Gynecologists (ACOG)
Includes data over the entire study period

| **Supplemental Table 2.** Maternal, perinatal, and structural characteristics of NTSV cesarean deliveries stratified by indication.^1^ | | | | | | | |
| --- | --- | --- | --- | --- | --- | --- | --- |
|  | **Overall**  N=3,629 | **Non-reassuring fetal status**  n=1,032 (28.4) | **Arrest of dilation**  n=948 (26.1) | **Arrest of descent**  n=739 (20.4) | **Failed induction**  n=343 (9.5) | **Abnormal placentation/other**  n=567 (15.6) | **p value** |
| Race/Ethnicity |  |  |  |  |  |  | <0.001 |
| NH White | 1,852 (51.0) | 479 (46.4) | 454 (47.9) | 435 (58.9) | 167 (48.7) | 317 (55.9) |  |
| NH Black | 431 (11.9) | 164 (15.9) | 111 (11.7) | 38 (5.1) | 49 (14.3) | 69 (12.2) |  |
| Hispanic | 545 (15.0) | 143 (13.9) | 182 (19.2) | 94 (12.7) | 61 (17.8) | 65 (11.5) |  |
| Asian | 341 (9.4) | 111 (10.8) | 83 (8.8) | 70 (9.5) | 31 (9.0) | 46 (8.1) |  |
| Other | 460 (12.7) | 135 (13.1) | 118 (12.4) | 102 (13.8) | 35 (10.2) | 70 (12.3) |  |
| Advanced Maternal Age | 1,431 (39.4) | 353 (34.2) | 364 (38.4) | 267 (36.1) | 159 (46.4) | 288 (50.8) | <0.001 |
| Partnered | 2,936 (80.9) | 819 (79.4) | 770 (81.2) | 643 (87.0) | 263 (76.7) | 441 (77.8) | <0.001 |
| Smoke exposure | 407 (11.2) | 109 (10.6) | 106 (11.2) | 86 (11.6) | 42 (12.2) | 64 (11.3) | 0.918 |
| Obese (BMI>30 kg/m2) | 2,206 (61.6) | 586 (57.9) | 642 (68.3) | 408 (55.8) | 254 (74.7) | 316 (56.3) | <0.001 |
| Gestational age (weeks) | 39.5 (1.1) | 39.7 (1.1) | 39.7 (1.0) | 39.6 (1.0) | 39.3 (1.2) | 38.7 (1.1) | <0.001 |
| Female neonatal sex | 1,547 (42.7) | 410 (39.7) | 404 (42.7) | 310 (41.9) | 162 (47.2) | 261 (46.0) | 0.053 |
| Birthweight (grams) | 3,441 (491) | 3,267 (464) | 3,557 (441) | 3,567 (419) | 3,379 (481) | 3,436 (601) | <0.001 |
| Polyhydramnios | 139 (3.8) | 24 (2.3) | 41 (4.3) | 23 (3.1) | 19 (5.5) | 32 (5.6) | 0.003 |
| Oligohydramnios | 55 (1.5) | 25 (2.4) | 11 (1.2) | 12 (1.6) | 3 (0.9) | 4 (0.7) | 0.041 |
| Placental abruption | 53 (1.5) | 37 (3.6) | 1 (0.1) | 4 (0.5) | 2 (0.6) | 9 (1.6) | <0.001 |
| Chorioamnionitis | 727 (20.0) | 203 (19.7) | 253 (26.7) | 202 (27.3) | 60 (17.5) | 9 (1.6) | <0.001 |
| Type of labor |  |  |  |  |  |  | <0.001 |
| Induction | 2,139 (58.9) | 671 (65.0) | 666 (70.3) | 430 (58.2) | 291 (84.8) | 81 (14.3) |  |
| Spontaneous | 1,044 (28.8) | 340 (32.9) | 279 (29.4) | 306 (41.4) | 49 (14.3) | 70 (12.3) |  |
| Chronic hypertension | 138 (3.8) | 22 (2.1) | 48 (5.1) | 18 (2.4) | 20 (5.8) | 30 (5.3) | <0.001 |
| Pregnancy induced hypertension | 859 (23.7) | 206 (20.0) | 278 (29.3) | 160 (21.7) | 130 (37.9) | 85 (15.0) | <0.001 |
| Preeclampsia |  |  |  |  |  |  | <0.001 |
| Preeclampsia without features | 158 (4.4) | 44 (4.3) | 49 (5.2) | 29 (3.9) | 23 (6.7) | 13 (2.3) |  |
| Preeclampsia with severe features | 365 (10.1) | 70 (6.8) | 129 (13.6) | 57 (7.7) | 70 (20.4) | 39 (6.9) |  |
| Pre-gestational diabetes | 61 (1.7) | 10 (1.0) | 14 (1.5) | 14 (1.9) | 8 (2.3) | 15 (2.6) | 0.104 |
| Gestational diabetes | 268 (7.4) | 44 (4.3) | 77 (8.1) | 45 (6.1) | 47 (13.7) | 55 (9.7) | <0.001 |
| Publicly insured | 403 (11.1) | 147 (14.2) | 109 (11.5) | 65 (8.8) | 46 (13.4) | 36 (6.4) | <0.001 |
| Weekday delivery (vs. weekend) | 2,229 (61.4) | 629 (60.9) | 570 (60.1) | 428 (57.9) | 193 (56.3) | 409 (72.1) | <0.001 |
| Day shift delivery (vs. night shift) | 1,132 (31.2) | 306 (29.7) | 270 (28.5) | 226 (30.6) | 64 (18.7) | 266 (47.0) | <0.001 |
| Attending practice model |  |  |  |  |  |  | <0.001 |
| Private | 2,948 (81.2) | 835 (80.9) | 763 (80.5) | 621 (84.0) | 289 (84.3) | 440 (77.6) |  |
| Academic | 453 (12.5) | 129 (12.5) | 105 (11.1) | 79 (10.7) | 47 (13.7) | 93 (16.4) |  |
| Unknown | 228 (6.3) | 68 (6.6) | 80 (8.4) | 39 (5.3) | 7 (2.0) | 34 (6.0) |  |
| Training of provider |  |  |  |  |  |  | <0.001 |
| Resident | 2,595 (71.6) | 795 (77.3) | 683 (72.1) | 528 (71.5) | 244 (71.3) | 345 (60.8) |  |
| Fellow | 69 (1.9) | 13 (1.3) | 15 (1.6) | 11 (1.5) | 9 (2.6) | 21 (3.7) |  |
| Attending | 959 (26.5) | 221 (21.5) | 249 (26.3) | 199 (27.0) | 89 (26.0) | 201 (35.4) |  |

NH = Non-Hispanic; BMI = body mass index

Data presented as N (%) or mean (standard deviation)

^1^Indications for cesarean delivery are as defined by the American College of Obstetricians and Gynecologists (ACOG)

Missing data (Obese: n=45, Neonatal sex: n=2, Birthweight: n=17, Insurance: n=1, Time of delivery: n=3, Training of provider: n=6)

Includes data over entire study period

| **Supplemental Table 3.** Unadjusted multinomial regression model estimates for the association of race and ethnicity and indication for NTSV cesarean delivery^1^ | | | | |
| --- | --- | --- | --- | --- |
|  | Indication for Cesarean | | | |
|  | Arrest of descent  RRR (95% CI) | Arrest of dilation  RRR (95% CI) | Failed induction  RRR (95% CI) | Non-reassuring fetal status  RRR (95% CI) |
| NH White | Ref | Ref | Ref | Ref |
| NH Black | **0.34 (0.21, 0.54)** | 1.15 (0.81, 1.63) | 1.37 (0.89, 2.11) | **1.72 (1.24, 2.39)** |
| Hispanic | 1.02 (0.72, 1.46) | 1.88 (1.36, 2.58) | 1.72 (1.15, 2.58) | 1.42 (1.02, 1.97) |
| Asian | 1.23 (0.81, 1.86) | 1.29 (0.86, 1.94) | 1.30 (0.77, 2.18) | **1.74 (1.18, 2.56)** |
| Other or unknown | 1.10 (0.77, 1.57) | 1.28 (0.90, 1.80) | 1.01 (0.63, 1.62) | 1.39 (0.99, 1.95) |
|  | | | | |

NTSV = Nulliparous Term Singleton Vertex, RRR=Relative Risk Ratio, CI=Confidence Interval, NH = Non-Hispanic

^1^Indications for cesarean delivery are as defined by the American College of Obstetricians and Gynecologists (ACOG)

Bolded text indicates significant results

Excludes two-month transition period

| **Supplemental Table 4.** Maternal, perinatal, and structural characteristics of study population pre and post intervention including two month transition period. | | |
| --- | --- | --- |
|  | **Pre-Intervention** N = 1,217 | **Post-Intervention** N = 2,206 |
| Race/Ethnicity, n (%) |  |  |
| NH White | 654 (53.7) | 1,114 (50.5) |
| NH Black | 136 (11.2) | 255 (11.6) |
| Hispanic | 167 (13.7) | 361 (16.4) |
| Asian | 118 (9.7) | 202 (9.2) |
| Other | 142 (11.7) | 274 (12.4) |
| Advanced Maternal Age, n (%) | 472 (38.8) | 887 (40.2) |
| Partnered, n (%) | 1,005 (82.6) | 1,767 (80.1) |
| Smoke exposure, n (%) | 152 (12.5) | 232 (10.5) |
| Obese (BMI>30 kg/m2), n (%) | 719 (59.9) | 1,359 (62.3) |
| Gestational age (weeks), Mean (SD) | 39.5 (1.1) | 39.5 (1.1) |
| Female neonatal sex, n (%) | 518 (42.6) | 937 (42.5) |
| Birthweight (grams), Mean (SD) | 3,439 (485) | 3,440 (497) |
| Polyhydramnios, n (%) | 46 (3.8) | 87 (3.9) |
| Oligohydramnios, n (%) | 13 (1.1) | 40 (1.8) |
| Placental abruption, n (%) | 13 (1.1) | 36 (1.6) |
| Chorioamnionitis, n (%) | 285 (23.4) | 404 (18.3) |
| Type of Labor, n (%) |  |  |
| Induction | 713 (58.6) | 1,306 (59.2) |
| Spontaneous | 367 (30.2) | 612 (27.7) |
| Chronic Hypertension, n (%) | 50 (4.1) | 85 (3.9) |
| Pregnancy Induced Hypertension, n (%) | 305 (25.1) | 505 (22.9) |
| Preeclampsia, n (%) |  |  |
| Preeclampsia without severe features | 48 (3.9) | 101 (4.6) |
| Preeclampsia with severe features | 122 (10.0) | 217 (9.8) |
| Pre-gestational diabetes, n (%) | 22 (1.8) | 37 (1.7) |
| Diagnosis of Gestational Diabetes, n (%) | 95 (7.8) | 162 (7.3) |
| Publicly Insured, n (%) | 129 (10.6) | 248 (11.2) |
| Weekday delivery (vs. Weekend), n (%) | 755 (62.0) | 1,331 (60.3) |
| Day Shift Delivery (vs. Night Shift), n (%) | 513 (42.2) | 535 (24.3) |
| Attending Practice Model, n (%) |  |  |
| Private | 881 (72.4) | 1,897 (86.0) |
| Academic | 150 (12.3) | 283 (12.8) |
| Unknown | 186 (15.3) | 26 (1.2) |
| Training of Provider, n (%) |  |  |
| Resident | 883 (72.7) | 1,570 (71.3) |
| Fellow | 27 (2.2) | 42 (1.9) |
| Attending | 305 (25.1) | 590 (26.8) |
| Cesarean delivery by indication, n (%)^1^ |  |  |
| Non-reassuring fetal status | 275 (22.6) | 700 (31.7) |
| Arrest of dilation | 482 (39.6) | 426 (19.3) |
| Arrest of descent | 277 (22.8) | 409 (18.5) |
| Failed induction | 11 (0.9) | 312 (14.1) |
| Other (ie. abnormal placentation) | 172 (14.1) | 359 (16.3) |

NH = Non-Hispanic; BMI = body mass index

Data presented as N (%) or mean (standard deviation)

^1^Indications for cesarean delivery are as defined by the American College of Obstetricians and Gynecologists (ACOG)

| **Supplemental Table 5.** Maternal, perinatal, and structural characteristics of study population pre and post intervention excluding two month transition period. | | |
| --- | --- | --- |
|  | **Pre-Intervention** N = 1,217 | **Post-Intervention** N = 2,206 |
| Race/Ethnicity, n (%) |  |  |
| NH White | 654 (53.7) | 1,114 (50.5) |
| NH Black | 136 (11.2) | 255 (11.6) |
| Hispanic | 167 (13.7) | 361 (16.4) |
| Asian | 118 (9.7) | 202 (9.2) |
| Other | 142 (11.7) | 274 (12.4) |
| Advanced Maternal Age, n (%) | 472 (38.8) | 887 (40.2) |
| Partnered, n (%) | 1,005 (82.6) | 1,767 (80.1) |
| Smoke exposure, n (%) | 152 (12.5) | 232 (10.5) |
| Obese (BMI>30 kg/m2), n (%) | 719 (59.9) | 1,359 (62.3) |
| Gestational age (weeks), Mean (SD) | 39.5 (1.1) | 39.5 (1.1) |
| Female neonatal sex, n (%) | 518 (42.6) | 937 (42.5) |
| Birthweight (grams), Mean (SD) | 3,439 (485) | 3,440 (497) |
| Polyhydramnios, n (%) | 46 (3.8) | 87 (3.9) |
| Oligohydramnios, n (%) | 13 (1.1) | 40 (1.8) |
| Placental abruption, n (%) | 13 (1.1) | 36 (1.6) |
| Chorioamnionitis, n (%) | 285 (23.4) | 404 (18.3) |
| Type of Labor, n (%) |  |  |
| Induction | 713 (58.6) | 1,306 (59.2) |
| Spontaneous | 367 (30.2) | 612 (27.7) |
| Chronic Hypertension, n (%) | 50 (4.1) | 85 (3.9) |
| Pregnancy Induced Hypertension, n (%) | 305 (25.1) | 505 (22.9) |
| Preeclampsia, n (%) |  |  |
| Preeclampsia without severe features | 48 (3.9) | 101 (4.6) |
| Preeclampsia with severe features | 122 (10.0) | 217 (9.8) |
| Pre-gestational diabetes, n (%) | 22 (1.8) | 37 (1.7) |
| Diagnosis of Gestational Diabetes, n (%) | 95 (7.8) | 162 (7.3) |
| Publicly Insured, n (%) | 129 (10.6) | 248 (11.2) |
| Weekday delivery (vs. Weekend), n (%) | 755 (62.0) | 1,331 (60.3) |
| Day Shift Delivery (vs. Night Shift), n (%) | 513 (42.2) | 535 (24.3) |
| Attending Practice Model, n (%) |  |  |
| Private | 881 (72.4) | 1,897 (86.0) |
| Academic | 150 (12.3) | 283 (12.8) |
| Unknown | 186 (15.3) | 26 (1.2) |
| Training of Provider, n (%) |  |  |
| Resident | 883 (72.7) | 1,570 (71.3) |
| Fellow | 27 (2.2) | 42 (1.9) |
| Attending | 305 (25.1) | 590 (26.8) |
| Cesarean delivery by indication, n (%)^1^ |  |  |
| Non-reassuring fetal status | 275 (22.6) | 700 (31.7) |
| Arrest of dilation | 482 (39.6) | 426 (19.3) |
| Arrest of descent | 277 (22.8) | 409 (18.5) |
| Failed induction | 11 (0.9) | 312 (14.1) |
| Other (ie. abnormal placentation) | 172 (14.1) | 359 (16.3) |

NH = Non-Hispanic; BMI = body mass index

Data presented as N (%) or mean (standard deviation)

^1^Indications for cesarean delivery are as defined by the American College of Obstetricians and Gynecologists (ACOG)
